# Beyond LDL: Understanding Triglyceride-Rich Lipoproteins to Tackle Residual Risk

**DOI:** 10.3390/jcm12123991

**Published:** 2023-06-12

**Authors:** Alejandro Gugliucci

**Affiliations:** Glycation, Oxidation and Disease Laboratory, Touro University California, Vallejo, CA 94592, USA; alejandro.gugliucci@gmail.com

For the past 30 years, statin therapy has been the cornerstone for the reduction in the risk of atherosclerotic cardiovascular disease (ASCVD). Low-density lipoprotein cholesterol (LDL-C) and ASCVD have been definitively linked in numerous investigations carried out throughout this time [1,2]. As a result of this relationship, drugs that lessen overall lifetime exposure to LDL-C have been successful in lowering the risk of ASCVD [1,2]. For the secondary prevention of cardiovascular disease (CVD), the European Society of Cardiology (ESC) reduced the target value for low-density lipoprotein cholesterol (LDL-C) from 1.8 mmol/L to 1.4 mmol/L in 2019. A recent paper set out to identify the clinical effects of the 2019 ESC/EAS dyslipidemia recommendations on lipid-lowering treatments and LDL-C target accomplishment rates in a current cohort of CAD patients enrolled in an ambulatory cardiac rehabilitation (CR) program [3]. The cohort was divided into a pre-Guideline 2019 group (A) and a post-Guideline 2019 group (B) to assess the impact of the publication of the guidelines. A total of 1320 patients were screened, leaving 875 for analysis. At discharge, more patients in group B were receiving the highest dosages of statins (20% vs. 9%, *p* < 0.0001) and were receiving ezetimibe in combination therapy (51% vs. 17%, *p* < 0.0001) when compared to those in group A, resulting in 53% of patients in group B achieving the LDL-C target of 1.4 mmol/L. Dyslipidemia and a history of smoking were found to be independent predictors for increased lipid-lowering medication [3]. Despite the effectiveness and safety of statin therapy, primary and secondary prevention adherence rates are still poor, at about 37% and 64%, respectively, and many patients are unable to meet LDL-C objectives [4]. For people with a history of ASCVD and LDL-C levels below 70 mg/dL or severe primary hypercholesterolemia with LDL-C levels above 100 mg/dL who are taking maximally tolerated statin therapy, new approaches are now available. The monoclonal antibodies Alirocumab and evolocumab can help with this, but their cost and necessity for biweekly injections prevent them from being widely used. Inclisiran, a siRNA treatment that silences PCSK9 expression and only needs to be injected twice a year to reduce LDL-C by 51%, was recently approved by the FDA. Bempedoic acid, a prodrug that targets ATP-citrate lyase in the cholesterol production pathway, is another drug that can be utilized in this population to reduce residual risk. Bempedoic acid is oral, affordable, and less expensive than PCSK9 inhibitors when it comes to lowering LDL-C; furthermore, when combined with ezetimibe, it lowers LDL-C by just a third as much as PCSK9 inhibitors [1,4]. To date, this is the state of the art of the LDL-centric approach. However, despite the efficiency and accessibility of statin drugs, and despite the use of PCSK9 inhibitors and other approaches cited above, ASCVD remains the primary cause of death worldwide. Indeed, is important to note that despite the great progress achieved with LDL-C treatment, many individuals on statin therapy continue to be at risk for ASCVD and are unable to achieve their goal LDL-C objectives. This residual risk, which may reach 50% after receiving effective statin treatment, results from immunological and/or lipid disturbances.

This is when triglycerides come to the forefront again. Triglyceride-rich lipoproteins (TRL) and their remnants have gained prominence in recent years in relation to residual risk. Triglycerides (TG) serve as indicators for the lipoproteins that transport them and, more particularly, for the byproducts of TRL degradation, which may have twice as much cholesterol as LDL. Ineffective intravascular TG metabolism and recapture result in the buildup of circulating TRL and their “remnants”, or partially lipolyzed derivatives. In addition to LDL, it is suspected that these cholesterol-rich residual particles also contribute to atherogenesis. Without a doubt, TG per se have no direct pathogenic effect on atherogenesis and are neutral from the perspective of pure chemistry. The correlation between plasma TG levels and other risk variables has prompted many researchers, but not all, to postulate that the relationship is confounded and probably not causal, even though epidemiological studies have long linked plasma TG levels to the risk of ASCVD. This view, however, changed when it was found that ASCVD was associated with differences in TG levels induced by heredity rather than low levels of high-density lipoprotein cholesterol (HDL-C). Additionally, clinical outcome studies conducted to assess the benefits of increasing HDL-C levels were unable to find a reduction in the risk of cardiovascular events. Although low HDL-C levels are now considered to be an indicator of ASCVD risk (mostly because they are a marker of TRL decreased turnover), they are not an efficient target for therapy. As a result, the management of increased TRL has emerged as the next promising lipid-lowering strategy to minimize ASCVD risk. The intravascular catabolism of chylomicrons and very low-density lipoproteins (VLDL) produces a variety of residual particles that have undergone partial lipolysis. The quantity and properties of these molecules in plasma are influenced by lipases, lipid transfer proteins, and the number of exchangeable lipoproteins. During their transit through the plasma, remnants may gain pathologic traits that help the development of ASCVD, such as increased cholesterol levels and the transfer of thrombogenic and inflammatory mediators. A wealth of knowledge regarding the role of TRL metabolism and the leftover particles produced as a result in atherogenesis has emerged over the past ten years, which has also encouraged the creation of new therapeutic targets. TRL fluxes are primarily controlled by lipoprotein lipase. The argument that delayed turnover or catabolism of TRL is more significant than excess production in the pathophysiology of hypertriglyceridemia is supported by the most recent findings. Heparan sulfate proteoglycans (HSPG), which are present on cell surfaces, are necessary for the transcytosis of the LPL molecule to the luminal face of capillaries. Glycosylphosphatidylinositol-anchored high-density lipoprotein-binding protein 1 (GPIHBP1) helps LPL become an active lipolytic enzyme and anchors it to the luminal surface of endothelial cells. LPL interacts with TRL and hydrolyzes TG to produce glycerol and free fatty acids. Fatty acids are used for oxidation in muscle and cardiac tissue or storage in adipocytes. Some of the fatty acids, or spillover fatty acids, remain in the bloodstream. Angiopoietin-like protein (ANGPTL) 3, 4, and 8 are the primary inhibitors of LPL, while insulin, apoCIII, AIV, and AV are the primary activators. In addition to the activation by apoCII and insulin and the inhibition by apoCIII, there is a tighter control of LPL activity in various tissues that makes it easier for the body to physiologically divide the TRL load according to its demands. Basically, during a fast, oxidative tissues such as the heart and skeletal muscle preferentially absorb lipids, while adipocyte storage is not preferred. On the other hand, LPL activity increases significantly in adipocytes after eating while decreasing in oxidative tissues. It is now clear that LPL is essential for the trafficking and partitioning of TG. While LPL activity in muscles reduces after eating, it increases in white adipose tissue (WAT) and increases during fasting in oxidative tissues. Although tremendous progress has been made in recent years, the mechanism controlling tissue-specific LPL activity throughout the fed–fast cycle is still largely unknown. For LPL activity to respond to constantly changing metabolic circumstances, tissue regulation is necessary. ANGPTL3, ANGPTL4, and ANGPTL8 have been identified as important tissue-specific regulators of lipolysis. In short, ANGPTL3 (secreted continuously from the liver) inhibits lipoprotein lipase in muscle and the heart during the postprandial period through an endocrine mechanism. In contrast, when fasting, ANGPTL4 released by adipocytes inhibits lipoprotein lipase in adipose tissue in a paracrine manner. The most recent hypotheses state that ANGPTL8 stimulates ANGPTL3 in an endocrine way to reduce LPL activity in the heart and skeletal muscle, while ANGPTL4 suppresses LPL activity in WAT by interacting with intracellular and circulating species. Fasting raises ANGPTL4 but lowers ANGPTL8, which lowers LPL activity in WAT and raises it in muscles, respectively. TG are consequently directed toward the muscles for oxidation. Eating, on the other hand, results in a drop in ANGPTL4 but an increase in ANGPTL8, which enhances LPL activity in the WAT while decreasing it in the muscles, directing circulation of TG to the WAT for storage. A growing body of research suggests that ANGPTL4 is linked to atherosclerosis. A recent article shows that ANGPTL4 expression was elevated in the endocardial adipose tissues (EAT) of patients with CAD for the first time, and it also shows that it is positively linked with IL-1 expression [5]. According to this research, ANGPTL4 in EAT may be a crucial mediator in reducing inflammation in atherosclerotic areas and may even serve as a biomarker for ASCVD. This finding could be very helpful in discovering how EAT metabolism speeds up the development of atherosclerosis and ASCVD. To better understand the connection between adipokines released by EAT and the development of atherosclerosis, additional fundamental and clinical investigations are required. The presence of ANGPTL3 and ANGPTL8 in muscle reduces LPL activity and increases TRL-triglyceride flow to adipose tissue during the postprandial state (when insulin levels are high). This is due to the important role ANGPTL3 plays in this process as supported by animal and human loss of function studies. Indeed, the ANGPTL3 monoclonal antibody evinacumab not only reduces TG but also LDL-C, probably because ANGPTL3 inhibits endothelial lipase levels [1,2,4]. The results from clinical trials demonstrated the efficacy and safety of evinacumab as an additional treatment for HoFH. In patients receiving maximally tolerated lipid-lowering treatment, evinacumab reduces LDL cholesterol levels by about 50%, and its mechanism is unrelated to the remaining LDLR activity. Additionally, evinacumab may possibly produce plaque regression, according to certain reports; however, this finding needs to be confirmed in randomized, placebo-controlled studies with a significant number of patients. Statins and PCSK9 inhibitors, which upregulate the LDLR pathway and are common lipid-lowering treatments, are ineffective or less effective in people who have two homozygous familial hypercholesterolemia (HoFH) null alleles. Evinacumab, on the other hand, decreases LDL-C levels without relying on LDLR activity. Therefore, it can be regarded as a key weapon in the arsenal of HoFH patients who failed to meet their minimal guideline-recommended LDL-C goals despite receiving several classes of lipid-lowering therapies and LDL apheresis, or it can be used as a substitute for patients who cannot receive apheresis or lomitapide [2,4]. The catabolic residues of TRL (remnants) are important pathogenic factors that explain the residual risk of ASCVD after optimal LDL-C levels have been obtained. The take-home messages are as follows: (1) TRL remnants can enter the arterial wall, and they frequently contain twice as much cholesterol as LDL. (2) Plasma triglycerides (TG) are used as a poor, yet useful surrogate marker of their presence. (3) The accumulation of circulating TRL and their residues is primarily due to impaired triglyceride catabolism. (4) Much progress has been heralded in the past decade, both at the physiological level of LPL regulation and via the quick passage from target to drugs in the case of apoCIII and ANGPTL3. The following objectives represent some of the numerous questions that remain unanswered and will undoubtedly drive active research in the coming years: understand the integration of the regulatory effects of apoCIII, apoAV, and ANGPTL on LPL activity; determine nutritional and hormonal factors that influence the activity of ANGPTL3, ANGPTL4, and ANGPTL8; determine the long-term effects if their control is pharmacologically interfered with.
